# From cheese whey permeate to Sakacin-A/bacterial cellulose nanocrystal conjugates for antimicrobial food packaging applications: a circular economy case study

**DOI:** 10.1038/s41598-020-78430-y

**Published:** 2020-12-07

**Authors:** Manuela Rollini, Alida Musatti, Daniele Cavicchioli, Daniele Bussini, Stefano Farris, Cesare Rovera, Diego Romano, Stefano De Benedetti, Alberto Barbiroli

**Affiliations:** 1 DeFENS, Department of Food, Environmental and Nutritional Sciences, Università degli Studi di Milano, Via Celoria 2, 20133 Milan, Italy; 2 ESP, Department of Environmental Science and Policy, Università degli Studi di Milano, Via G. Celoria 2, 20133 Milan, Italy; 3 SSCCP, Paper Area, INNOVHUB - Stazioni Sperimentali per l’Industria S.r.l., Via G. Colombo 83, 20133 Milan, Italy

**Keywords:** Sustainability, Food microbiology, Nanoparticles, Antibiotics

## Abstract

Applying a circular economy approach, this research explores the use of cheese whey permeate (CWP), by-product of whey ultrafiltration, as cheap substrate for the production of bacterial cellulose (BC) and Sakacin-A, to be used in an antimicrobial packaging material. BC from the acetic acid bacterium *Komagataeibacter xylinus* was boosted up to 6.77 g/L by supplementing CWP with β-galactosidase. BC was then reduced to nanocrystals (BCNCs, 70% conversion yield), which were then conjugated with Sakacin-A, an anti-*Listeria* bacteriocin produced by *Lactobacillus sakei* in a CWP based broth. Active conjugates (75 Activity Units (AU)/mg), an innovative solution for bacteriocin delivery, were then included in a coating mixture applied onto paper sheets at 25 AU/cm^2^. The obtained antimicrobial food package was found effective in reducing *Listeria* population in storage trials carried out on a fresh Italian soft cheese (named “stracchino”) intentionally inoculated with *Listeria*. Production costs of the active material have been mainly found to be associated (90%) to the purification steps. Setting a maximum prudential 50% cost reduction during process up-scaling, conjugates coating formulation would cost around 0.89 €/A4 sheet. Results represent a practical example of a circular economy production procedure by using a food industry by-product to produce antimicrobials for food preservation.

## Introduction

Bacteria are beneficial production platforms for a wide range of biomolecules and, combined with agro-food residues degradation, have the potential to establish examples of production processes in the frame of the emerging bio-based economy. Cheese whey permeate (CWP) represents the by-product of whey ultrafiltration for protein recovery: it is highly perishable, has high disposal costs and serious environmental impact, nevertheless retaining valuable milk components at low cost^1^. Technology improvement in ultrafiltration has allowed to obtain a variety of whey proteins concentrates (WPCs) for different purposes, such as nutritional supplement, infant formula and sports beverages^2^. The combined effect of nutritional properties of WPCs and their cost-efficient production will boost their supply over the next years. Because of an expected increase in demand for WPCs, the availability of CWP is expected to grow at the same pace, representing a valuable resource for circular economy activities^3^.

Bacterial cellulose (BC) is a natural biomaterial industrially produced by acetic acid bacteria belonging to the *Komagataeibacter* genus (mainly from *K. xylinus*), through static fermentation of different carbon sources (i.e. glucose, fructose, sucrose, glycerol)^4^. Its peculiar physico-chemical properties, including high crystallinity, nanofibrous network structure, purity, degree of polymerization, high water absorption and holding capacity, superior tensile strength, good biocompatibility, resistance to chemical and heat shock, lack of toxicity, easy sterilization, selective porosity, are exploited in many industrial fields such as biomedical (manufacturing of artificial blood vessels, skin, cornea, cartilage and bone)^5^, cosmetics (active ingredients delivery systems as facial masks)^6^, textile, high quality paper production and food as thickening and stabilizing agent^7^. Nevertheless, its industrial production and commercialization at large scale are still actually hampered by high fermentation costs, low productivity and expensive culture media. To address this issue and enhance process sustainability, different studies have focused on using agro and industrial wastes as fermentation feedstock, including cheese whey and its derivatives^8,9^. Relatively few studies have aimed at evaluating whey as a substrate for BC production, mainly because BC-producing strains lack the enzymes involved in the use of lactose as carbon source. Thus a hydrolytic pretreatment (acid or enzymatic hydrolysis of lactose) or the use of β-galactosidase recombinant strains are often used. Nevertheless, yields are still low compared to other agro-industrial wastes and further studies are needed for the bioprocess improvement^10^.

BC has the potential to act as a valuable building block for novel materials^11,12^, including the preparation of bacterial cellulose nanocrystals (BCNCs), obtained by chemical modification of BC. BCNCs have potential applications across several industrial sectors and enable the development of innovative materials, as well as the enhancement of conventional materials properties. BCNCs have attracted much attention due to their renewable nature, biodegradability and biocompatibility. All these features have been extensively reviewed^13–15^. In the field of food sciences, research is focusing on the development of new packaging materials, where BCNCs are added as nanofiller in papers and boards or are included in coatings for plastic matrices to improve functional properties. In the same field, an interesting potential application of BCNCs involves the use of cellulose nanocomposites to extend the shelf life and enhance the quality of perishable foods, serving as a carrier of active substances (e.g. antimicrobials), in active packaging^16^.

Active packaging is defined as “a mode of packaging in which the package, the product and the environment interact to prolong shelf life or enhance safety or sensory properties, while maintaining the quality of the food product”^17^. Active packaging positively employs the mass transfer phenomena that take place in polymeric materials: permeation, migration and sorption^18^. Few active packaging systems are commercially available nowadays, despite an extensive research to limit foodstuffs deterioration with synthetic and naturally-occurring antimicrobial compounds, such as organic acids/anhydrides, parabens, inorganic gases, metals, chelating agents, essential oils, enzymes, etc.^19^.

Among antimicrobial agents, those generated by bacteria (bacteriocins) are raising increasing interest due to their greater potential over other antimicrobials^20^. Bacteriocins are ribosomally synthetized peptides produced by lactic acid bacteria (LAB) as a first line of defense against competitors^21,22^. Despite nisin remains the only bacteriocin approved as such for use as a preservative in food, LAB have attained a “Generally Regarded as Safe” (GRAS) status and bacteriocins in general have been shown to be safe for consumption because of their safe origin^23^. Bacteriocins are completely digested in the gastrointestinal tract, are resistant to the common thermal treatments for pasteurization or in some cases even sterilization, and are 10^3^–10^6^ times stronger than several known antimicrobials (ampicillin, penicillin, chloramphenicol…) against pathogenic microorganisms, such as *Listeria monocytogenes*^24–26^. The use of bacteriocins in food preservation may also offer the benefit of meeting the consumers request for minimally processed food containing natural additives^27^.

According to Cotter et al*.*^21^, bacteriocins are classified based on their structure. Among them, class I and class II include small and thermostable peptide: in class I are allocated post translationally modified peptides, whereas class II peptides contain only standard amino acids. Sub-group class IIa bacteriocins share high sequence similarity (40–60%), a conserved cationic and amphipathic architecture, and the presence of the consensus sequence YGNGV. They are strong inhibitors of *L. monocytogenes*, but their antimicrobial spectrum also includes species of the genera *Enterococcus*, *Carnobacterium*, *Lactobacillus*, *Leuconostoc*, *Pediococcus* and *Clostridium*^22^.

Sakacin-A (UniProtKB entry P0A310) is a class II A bacteriocin of 41 amino acids, molar mass of 4308.9 Da and pI of 9.32 produced by *Lactobacillus sakei*^28^. Compared to other class IIa bacteriocins, which act mainly by permeabilizing *Listeria* membrane, Sakacin-A is also able to induce cell wall degradation of *Listeria* by a lytic activity^29^. In spite of some examples in the meat sector (see *L. sakei* as a food starter) and as a food preservative, the use of Sakacin-A is still in its infancy, with in situ applications (i.e. adding bacteriocin-producing bacteria directly on food product) being the only authorized approach, while ex situ applications (the addition of semi-purified or purified peptides) probably being the most promising alternative solutions^30^. Sakacin-A incorporated in pullulan films has been first proven to be effective in the reduction of *L. monocytogenes* in intentionally inoculated sliced turkey breast by Trinetta et al*.*^31^ when storage lasted 3 weeks under refrigeration temperature. Barbiroli et al*.*^32^ set up a gelatin-based coating containing Sakacin-A. When the coating was spread on the polyethylene side of a coated paper wrapping, the active packaging was effective in controlling *L. innocua* development in thin-cut veal meat slices intentionally inoculated. More recently, Mapelli et al*.*^33^ adsorbed Sakacin-A in a cellulose nanofibers pad to be used as an active antimicrobial mat (or separator) in direct contact with foods; the active pad was able to reduce *L. innocua* population of about 2.5 log cycles in intentionally inoculated smoked salmon fillets after 4 weeks storage at refrigeration temperature.

Under a general point of view, application of bacteriocins at an industrial level is limited by their low yield of production^34,35^, that requires to combine different purification techniques to obtain a pure molecule (mainly salt precipitation, ultrafiltration, and traditional or high pressure liquid chromatography). Obviously the purification procedure strictly depends on the molecular features of the bacteriocins, and a “universal” purification procedure, suitable for all classes of bacteriocins, is unlikely to be set up. Purification possibilities have to be evaluated according to the target of bacteriocin application, in order to find the best compromise among level of purity, recovery, yield, and duration of the process^34,36^.

In the frame of applying a bio-based circular economy approach, this study aims to explore the use of CWP as cheap substrate for the production of Sakacin-A and BCNCs. The two compounds will be applied in an active packaging formulation to enhance the food safety in ready-to-eat products, thus providing a sustainable path to relevant alternative and industrial use of this residue. To this purpose, we propose an innovative procedure to obtain a preparation enriched in Sakacin-A, exploiting its binding capacity towards BCNCs. Sakacin-A/BCNCs conjugates have been applied in coating formulation for food packaging material. The efficacy of the antimicrobial active packaging has been proven for an Italian fresh soft cheese (named “stracchino”), by applying the circular economy concepts expressed by de la Caba et al*.*^37^, that is: using a food industry by-product to produce antimicrobials for food preservation. Finally, the proposed approach has been examined in terms of economic feasibility, through the computation of lab-scale production costs and simulating cost savings ensuing from a process upscaling. Consumer acceptance of the proposed antimicrobial packaging has also been discussed as a key-factor to achieve a comprehensive economic sustainability, in light of a possible use by the food industry.

## Results and discussion

### BC production, purification and characterization

**Figure 1:**
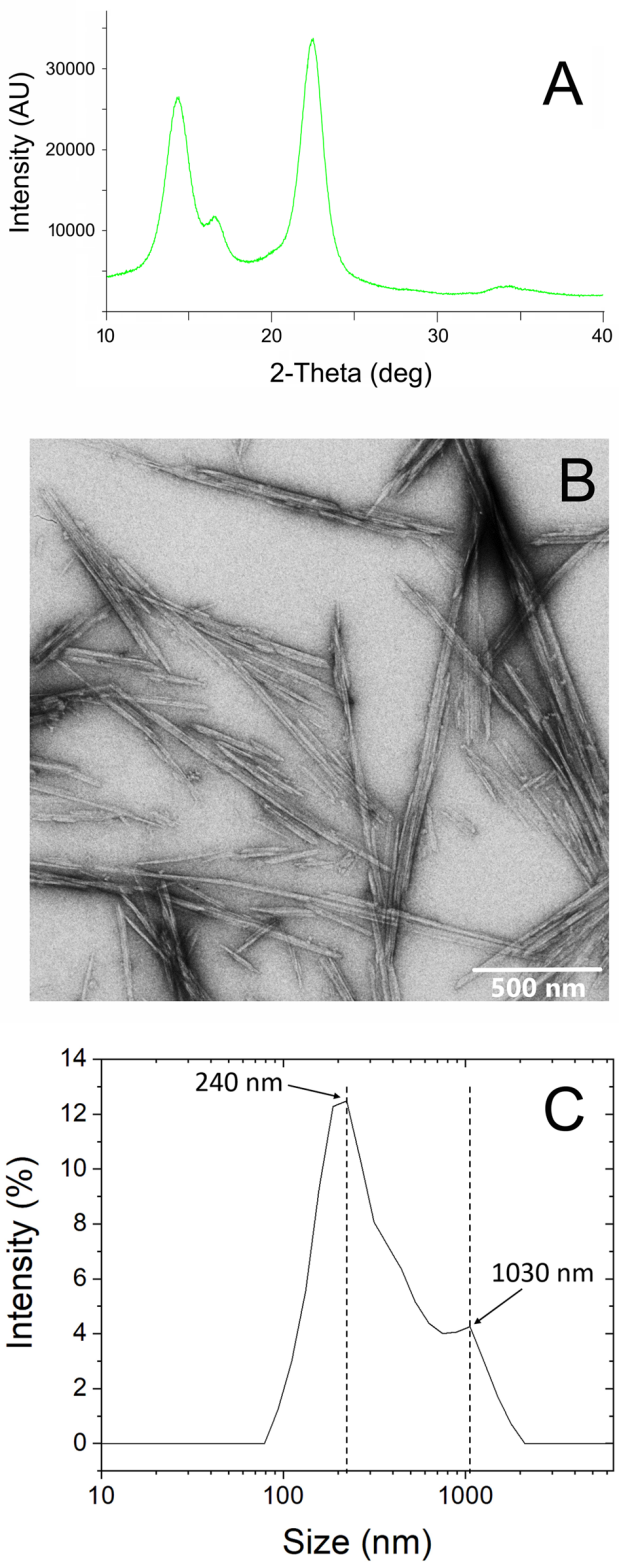
BCNCs production and characterization. (**A**): X-ray diffraction analysis of BC produced by *K. xylinus* DSM 2325; (**B**) transmission electron microscopy images of needle-like BCNCs; (**C**) dynamic light scattering size distribution analysis of BCNCs.

The β-galactosidase activity in acetic acid bacteria producing BC is very low, thus leading to negligible yields of BC when lactose is the primary carbon source^38^. Hence, in this study we implemented a hydrolytic pretreatment for the production of BC from CWP. BC production was carried out in CWP in presence of a minimal amount of β-galactosidase (0.5 U/mL, 30 °C). Using this strategy, a slow lactose hydrolysis was obtained, thus subjecting the cells to a gradual increasing concentration of sugars. *K. xylinus* DSM 2325 reached the highest BC yield, 6.77 ± 0.14 g/L; this value is slightly higher than those obtained in literature using cheese whey as fermentation feedstock^38^. Structural organization of obtained BC was analyzed via X-ray diffraction (XRD). Figure 1A shows the XRD diffractogram obtained from the freeze-dried BC, with the two main peaks at 2θ ≈ 22.5° (d ≈ 3.95 Å) and 2θ ≈ 14.3° (d ≈ 6.17 Å). A third smaller peak is also present at 2θ ≈ 16.6° (d ≈ 5.33 Å). This pattern is associated with the randomly oriented triclinic crystalline form (Iα) of cellulose, the dominant form in bacteria^39^. The crystallinity index (CI) of BC was estimated according to the peak height method developed by Segal et al.^40^ and calculated to be as high as 82%, in line with the values found by Sacui et al*.*^41^ using a combined CPMAS-NMR/WAXS spectroscopy approach.

### Characterization of bacterial cellulose nanocrystals (BCNCs)

The yield of the acid hydrolysis used to obtain BCNCs was 70 ± 4%. BCNCs dispersed in water exhibited ζ-potential values of − 33.0 ± 0.2 mV. This value is in line with what previously reported by Vasconcelos et al.^42^ and Yan et al.^43^ using the same substrate and a hydrolytic procedure. In particular, they reported a ζ-potential value of about − 26 and − 34 mV, respectively. ζ-potential has a practical importance because it reflects the high stability of the BCNCs colloidal dispersion in water^43^. The ζ-potential value measured for the BCNCs obtained in this study can be explained in consideration of the esterification of the hydroxyl group on the C6 induced by the sulfuric acid during the hydrolysis process, which led to the formation of sulfate groups (–SO_3_^−^). The morphology of BCNCs arises from the hierarchical structure of BC, organized in flat ribbons with rectangular cross-sections that also include the crystal domains (known as nanocrystals). The highly ordered crystalline domains are characterized by a parallel configuration of cellulose chains, according to the specific hydrogen bonding pattern typical of allomorph Iα.

Morphology and size distribution of BCNCs were investigated by transmission electron microscopy (TEM) and dynamic light scattering (DLS), respectively. Figure 1B displays BCNCs images acquired by TEM. From a morphological point of view, the hydrolysis process mediated by sulfuric acid generated a needle-like morphology of nanocrystals with an average length of 375.0 ± 41.5 nm and width of 21.6 ± 7.5 nm, in line with the values found by Singhsa et al*.*^44^. Because of the random orientation of BCNCs and the relatively high concentration of the water dispersion, partial overlapping between nanocrystals was also observed, most likely driven by intermolecular hydrogen bonding.

DLS experiments suggested that the hydrolysis process yielded a polydisperse size distribution of BCNCs, as also observed by other authors for acid-hydrolyzed BC^45–47^. As shown in Fig. 1C, it was possible to observe two main peaks, the biggest in intensity centered at about 240 nm and the less intense at 1030 nm, respectively. The broad appearance of the DLS pattern confirms a heterogeneous size distribution that gradually shifts from the first, more intense peak to the second one. In other words, while most particles had a mean size slightly above 200 nm, the dispersions apparently also included particles of bigger size, up to approximately 1 μm. The discrepancy between TEM and DLS results lies in the inherent differences between the two techniques. While TEM provides absolute values, DLS is based on the light scattered by the dispersed particles and the final value can be affected by a number of factors, such as the shape of the particles (the Stokes–Einstein relationship is used to calculate the hydrodynamic radius of particles considering them as spheres) and particle–particle interactions that may affect the particles motion. The DLS values obtained in this work thus reflect both the actual size of BCNCs, in line with previous works^48^, and possibly overlapping nanocrystals, confirming what was also observed by TEM.

### Sakacin-A production and enrichment

**Figure 2:**
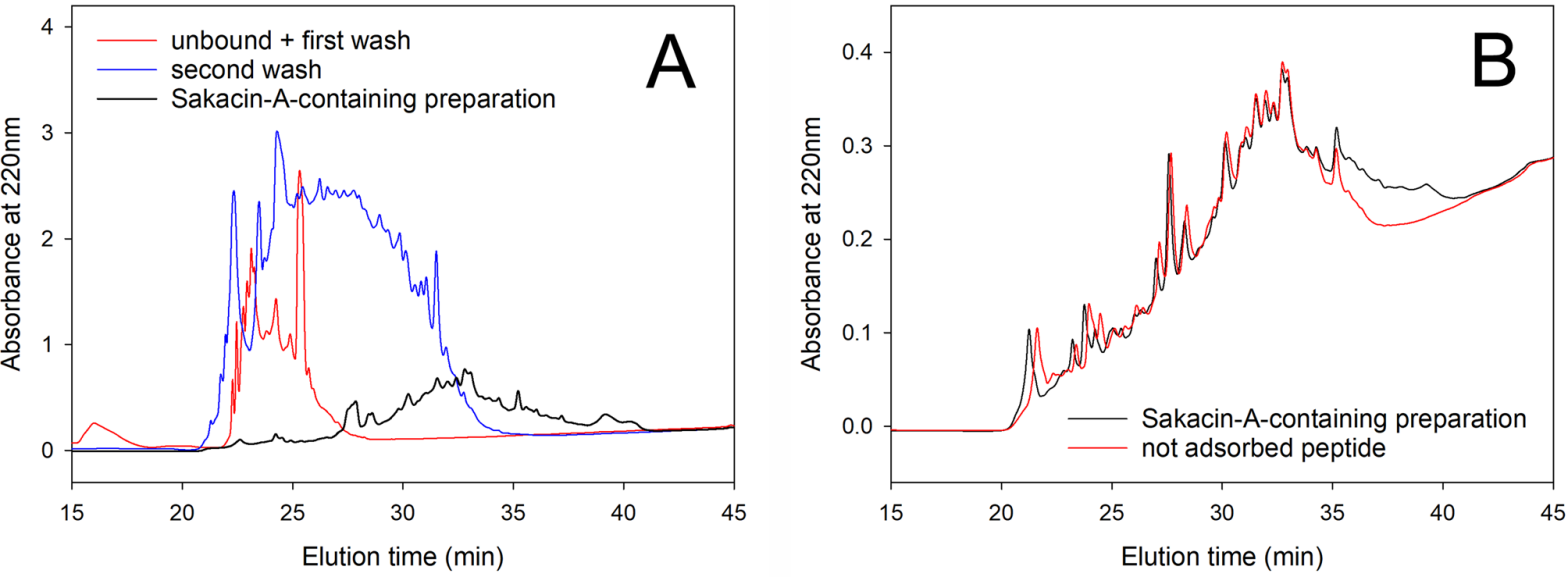
Sakacin-A purification and conjugation with BCNCs. (**A**) enrichment procedure of Sakacin-A. RP-HPLC chromatograms of unbound fraction plus the first wash (red), second wash (blue) and Sakacin-A-containing preparation (black); (**B**) conjugation of Sakacin-A to BCNCs. RP-HPLC chromatograms of Sakacin-A-containing preparation (black), and supernatant fraction residual after conjugation (i.e. not adsorbed peptides) (red). The linear increase of the baseline over time was due to the spectroscopic properties of ACN.

A Sakacin-A-containing preparation was obtained by a reverse-phase traditional chromatography from *L. sakei* DSMZ 6333 centrifuged cultures. The enrichment procedure takes advantage from the amphipathic nature of Sakacin-A that, in its structure, opposes to a hydrophilic N-terminal a highly hydrophobic C-terminal, the latter responsible for the overall hydrophobic behaviour of the bacteriocin. Small polar molecules, including salts, and the majority of peptides were removed in the unbound fraction and in the washing step (acetonitrile, ACN 25%), whereas the amphipathic Sakacin-A was contained in the fraction eluted at 40% ACN (Fig. 2A). No further activity was detected in fractions eluted at ACN concentration higher than 40%. Volatile solvent was completely removed from the Sakacin-A-containing preparation at first by evaporation in Rotavapor, followed by lyophilization. No loss of activity was observed during evaporation of the solvent. The quantitative characterization of the procedure is reported in Table 1. As shown, the Sakacin-A-containing preparation contains only a small fraction of the total protein amount present in the centrifuged cultures, but more than half of the total activity. Overall, the set-up procedure allows a straightforward enrichment of the antimicrobial activity with a tenfold increase of the specific activity and a total recovery of 620667 Activity Units (AU) from the 7 L culture, corresponding to a 53% yield.

**Table 1:** Purification table of Sakacin-A-containing preparation. Data are representative for a single chromatographic procedure.

Purification step	Volume (mL)	Total protein (mg)	Total activity (AU)	Specific activity (AU/mg protein)	Yield (%)
Centrifuged culture	750	4516	126085	27.9	100
Unbound fraction + first wash (TFA 0.1%)	850	2706	ND	–	–
Second wash (ACN 25%, TFA 0.1%)	100	1424	ND	–	–
Elution (ACN 40%, TFA 0.1%)	100	244	66500	272	53

*ND* not detected.

Many protocols have been proposed for Class IIa bacteriocins purification, mainly based on salt precipitation followed by various combinations of chromatographic approaches (ion-exchange, reverse-phase HPLC, etc.), however, most of them are aimed at the production of highly purified molecule with consequently low yields and high time-consumption^49^. On the contrary, purification for food applications requires a balance between the degree of purification and the related costs, keeping a close eye on the safety issues. In Mapelli et al*.*^33^ an ammonium sulphate precipitation was applied to obtain an enriched Sakacin-A extract: although the specific activity was almost the same (256 AU/mg of total protein, respect to the 272 AU/mg here obtained), the total yield of the enrichment procedure provided a lower recovery (25 vs. 53% here obtained). Finally, it is remarkable that the Sakacin-A-containing preparation here obtained is virtually salt free and, after freeze-drying, retains a weak acidic pH, thus has suitable characteristics to be conjugated with BCNCs as reported below.

### Sakacin-A/BCNCs conjugates

Sakacin-A has a positively charged N-terminal (calculated pI 9.31). When the Sakacin-A-containing preparation is mixed with the BCNCs suspension, the bacteriocin spontaneously binds to the negatively charged nanocrystals forming electrostatically-stabilized conjugates, easily recovered by centrifugation. The ratio of conjugation has been calculated in 75 AU/mg by measuring the residual activity in the supernatant (not adsorbed fraction). To get an insight on the pattern of peptide adsorbed by BCNCs, the not adsorbed fraction was analyzed by RP-HPLC in comparison to the enriched Sakacin-A preparation (Fig. 2B). Interestingly, among the peptides present in the preparation, only a small population of them, that include the bacteriocin, is adsorbed onto the BCNCs, thus the conjugation step allowed a further purification of the bacteriocin. The obtained conjugates were found stable upon incubation in neutral and mild acidic solution (pH 5) (i.e. the bacteriocin is not released in these conditions), but Sakacin-A completely dissociated from BCNCs in alkaline condition (pH 11), that reverses the charge of the bacteriocin, or in saline solution (≥ 20 mM NaCl).

The evidence of a “reversibility” of the association is of primary importance, since the bacteriocin, to exert its activity, should be able (1) to migrate from the antimicrobial device to the food and (2) to be free to interact with *Listeria* membrane. In other words, the conjugation of Sakacin-A with BCNCs mimics a step of cation-exchange purification, exploiting the huge surface/volume ratio typical of nanosized structure, where the “elution” is triggered directly by the contact with the food.

### Production and characterization of coated paper samples

Four coating formulations were used for the production of coated paper samples: Sakacin-A/BCNCs conjugates, BCNCs, Sakacin-A, and hydrohypropylcellulose (HPC), the latter used as control and thickener for the other formulations. Antimicrobial preparations were spread achieving about 25 AU/cm^2^, as calculated from the increment of grammage (Table 2).

**Table 2:** Properties of the coated paper samples.

Properties	Paper substrate	HPC	Sakacin-A	BCNCs	Sakacin-A/BCNCs conjugates
Grammage^1^ (g/m^2^)	45.2 ± 0.1^a^	51.0 ± 0.9^b^	50.6 ± 0.8^b^	48.8 ± 0.3^c^	48.3 ± 0.4^c^
Thickness^1^ (µm)	56.9 ± 2.0^a^	63.7 ± 1.3^b^	63.3 ± 1.6^b^	63.7 ± 1.1^b^	64.1 ± 1.4^b^
Surface roughness Bendtsen^1^ (mL/min)	133 ± 14^a^	104 ± 21^b^	116 ± 25^a^	123 ± 24^ab^	174 ± 31^c^
Tensile strength^1^ (N/m)	3157 ± 276^a^	3740 ± 106^b^	3705 ± 212^b^	3548 ± 207^bc^	3408 ± 171^ac^
Coated activity (AU/cm^2^)^2^	–	–	29.5	–	23.2

Samples marked with the same superscript letter are not significantly different (*p* < 0.05).

^1^Number of measured samples: grammage, 4; thickness, 20; surface roughness, 20; tensile strength, 8.

^2^Coated activities were calculated from the grammage considering the dry weight and the activity content of the coating formulations.

Physical–mechanical properties of the paper were marginally affected by the coating made of Sakacin-A/BCNCs conjugates (Table 2). Sakacin-A/BCNCs conjugate coated sample had a higher surface roughness compared to paper substrate and all reference samples. Tensile strength of BCNCs coated reference sample increased with respect to the paper substrate, because of the effect of BCNCs, and a similar apparent effect was found in the Sakacin-A/BCNCs conjugate coated sample. HPC showed higher potential in increasing tensile strength than BCNCs in the conditions of coating trials.

**Figure 3:**
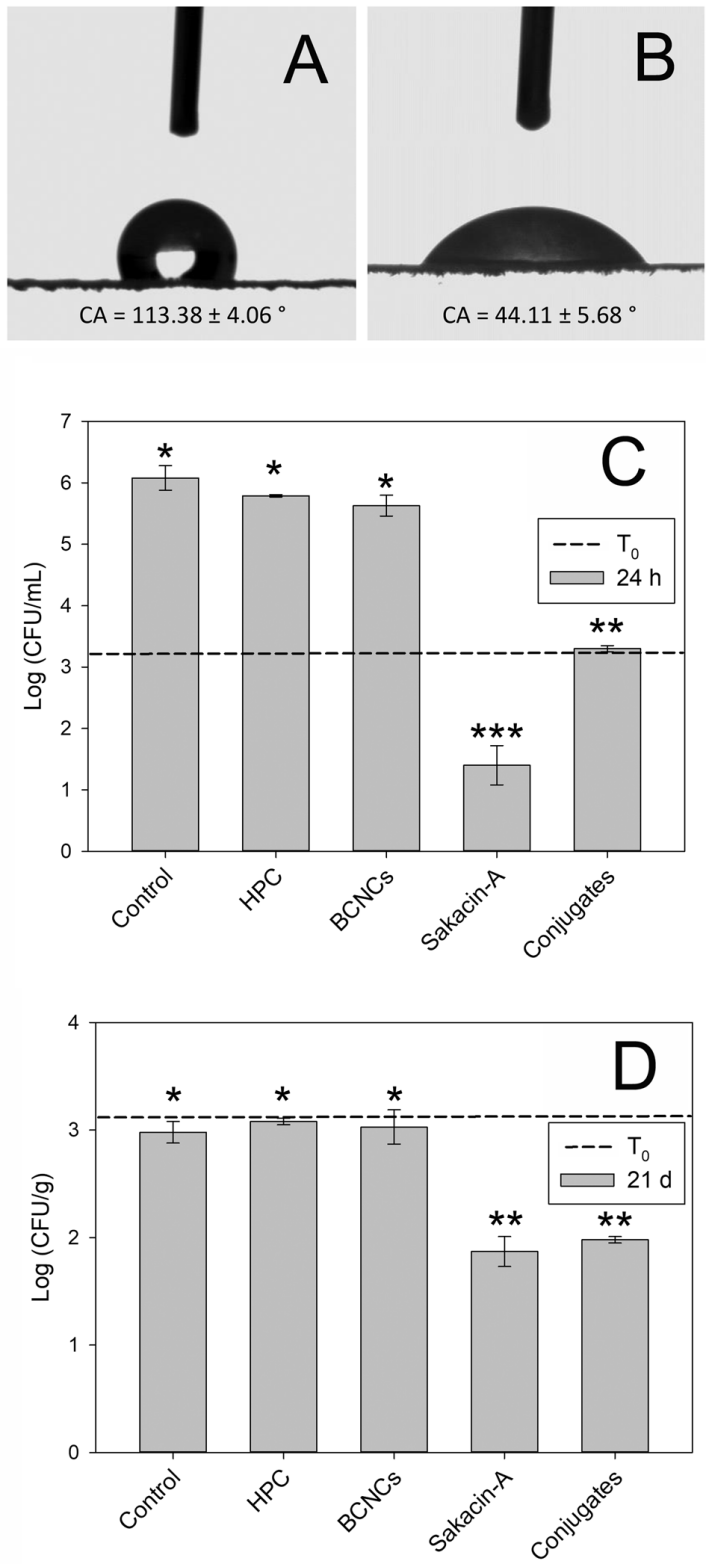
Active packaging. (**A**,**B**): Contact angle (CA) of paper substrate (**A**) and of Sakacin-A/BCNCs coated paper (**B**). (**C**): in vitro quantitative assessment of antimicrobial activity of the coated paper samples. Concentration (Log CFU/mL) of a *L. innocua* cell suspension at t_0_ (dashed line) and after 24 h incubation at 37 °C in absence (control) or in contact with HPC reference, BCNCs reference, Sakacin-A reference, and Sakacin-A/BCNCs conjugates coated paper samples. Samples marked with the same number of stars are not significantly different (*p* < 0.05). (**D**) in vivo quantitative assessment of antimicrobial activity of the coated paper samples. *L. innocua* population (Log CFU/g) in samples of “stracchino” soft cheese intentionally inoculated (dashed line) and then stored for 21 days at 5 ± 1 °C in absence (control) or in contact with HPC reference; BCNCs reference, Sakacin-A reference, and Sakacin-A/BCNCs conjugates coated paper samples. Samples marked with the same number of stars are not significantly different (*p* < 0.05).

Surface wetting analysis showed a significant decrease of the contact angle in the Sakacin-A/BCNCs conjugate coated paper compared to the paper substrate, due to the more polar nature of the coating formulation. The higher wettability of the coated samples was primarily due to spreading rather than to absorption of the water droplet across the material thickness (Fig. 3A,B). Interestingly, the increased wettability positively affects the migration process of the bacteriocin from the packaging to the food, as well as the presence of physiological fluids of foods, usually high in ionic strength, which allow both the dissociation of Sakacin-A from BCNCs, and its diffusion from the packaging to the surface of the product. Finally, none of the coated samples had grease resistance, as the Kit Test value result was below 3 for all the samples.

### In vitro antimicrobial activity of the coated paper samples

The Minimum Inhibitory Concentration (MIC) was 10 AU/mL, both for the Sakacin-A-containing preparation and for the conjugates. Qualitative assessment of the antimicrobial activity with the agar disk diffusion method evidenced that *L. innocua* growth was inhibited both by paper samples coated with the Sakacin-A/BCNCs conjugates (diameter of inhibition halo: 21.8 ± 0.5 mm) and with the Sakacin-A reference (diameter of inhibition halo: 26.7 ± 1.5 mm). This difference, that in part could be also due to the slightly lower AU coated when dealing with the conjugate reference, suggests that in a time-limited experiment (16 h incubation) paper samples coated with only Sakacin-A were more effective in limiting *Listeria* growth rather than the paper coated with the Sakacin-A/BCNCs conjugates. No microbial inhibition was instead evidenced neither with paper sample coated with HPC nor when coated with BCNCs, thus confirming that the antimicrobial activity was attributable exclusively to Sakacin-A.

In vitro quantitative assessment of the antimicrobial activity evidenced that neither the contact with HPC nor with the BCNCs alone caused reduction of *L. innocua* cell (Fig. 3C): growth indeed increased from 3 to 5.71 ± 0.14 Log CFU/mL. On the contrary, culture incubation in presence of paper coated with Sakacin-A alone decreased *Listeria* population from 3 to 1.40 ± 0.32 Log CFU/mL; in the presence of the conjugates-coated papers, antimicrobial activity was weaker, however it kept cell concentration at its initial value (3.30 ± 0.05 Log CFU/mL), limiting *Listeria* growth.

In a previous work^33^, similar trials were carried out with a Sakacin-A preparation incorporated (not coated) into a pad matrix made of cellulose nanofibers (CNF) at a double concentration respect to what used here (50 vs. 25 AU/cm^2^), evidencing a 2-log cycle reduction (from 6 to 4 Log CFU/mL) of *Listeria* growth compared to 1.6 Log reduction here obtained, confirming its antimicrobial efficacy.

These results strengthened the qualitative trials, as Sakacin-A antimicrobial activity resulted stronger when the bacteriocin was coated alone rather than as conjugate with BCNCs, even if again a slightly lower bacteriocin concentration was found to be coated in the conjugate reference. This behaviour may be related to the bacteriocin mechanism of action against the target cell: Sakacin-A acts primarily by binding its positively charged N-terminal region to a specific receptor on the *Listeria* membrane and then the hydrophobic C-terminal half inserts in the membrane, leading to pore formation and cell death^22,33^. A lower antimicrobial activity of the conjugates may be attributable to a slow release of Sakacin-A from nanocrystals, thus reducing the extent of the antimicrobial activity in time-limited experiments.

### Antimicrobial activity of coated paper on “stracchino” soft cheese

Coated paper samples were evaluated for their antimicrobial activity on real food in samples of “stracchino” soft cheese intentionally inoculated with *L. innocua* (Fig. 3D). Results showed a 1 log cycle reduction of *Listeria* population after 21 days of incubation in samples packaged using paper coated with either Sakacin-A/BCNCs conjugates or Sakacin-A only, when compared to the population inoculated at t_0_ (respectively 1.98 and 1.87 vs. 2.98 Log CFU/g). The Total Aerobic Count (TAC) of cheese was 3.92 ± 0.14 Log CFU/g at t_0_, and increased in all samples to reach around 5.7 Log CFU/g at 21 d, highlighting the specificity of Sakacin-A antimicrobial activity against *Listeria*.

Unlike in vitro trials, these experiments demonstrated that the antimicrobial activity of the paper samples coated with Sakacin-A and Sakacin-A/BCNCs conjugates was similar. This behavior may be due to the longer time of incubation here observed, which makes up for diffusional effects.

Similar results were obtained by Barbiroli et al*.*^32^, which developed a Sakacin-A active paper by coating a polyethylene-coated paper with a crude Sakacin-A extract, tested on thin-cut veal meat slices inoculated with *Listeria*.

### Cost estimation of active formulations (lab-scale)

Given the potential interest of the active material for food packaging purposes, an estimation of its production cost at laboratory scale is provided. The estimation procedure is based on data from previous sections and follows the methodology used in Musatti et al.^3^, based on cost minimization criteria. As a first step, production costs for each component of the active coating formulations were estimated. Such values are expressed as €/10^6^ AU for Sakacin-A (production and purification), and in €/g for both BC (production and purification) and BCNCs (yield from BC and purification costs). Such data, along with the assumptions made for their calculations, are reported in Table 3.

**Table 3:** yields and production costs for Sakacin A, bacterial cellulose (BC) and cellulose nanocrystals (BCNCs).

Production	Stage	Yields and production costs	Share on total production cost (%)
Sakacin-A	Cost of the culture medium (€/L)	1.53	
	Fermentation yield (10^6^ AU/L)	0.27	
	Production cost (€/10^6^ AU)	5.67	9
	Purification cost (€/10^6^ AU)	60	91
Total cost Sakacin-A: production + purification (€/10^6^ AU)		65.67	100
Bacterial cellulose (BC)	Cost of the culture medium (€/L)	0.18	
	Fermentation yield (g/L)	6	
	Production cost (€/g)	0.03	12
	Purification cost (€/g)	0.23	88
Total cost: production + purification (€/g)		0.26	100
Cellulose nanocrystals (BCNCs)	Conversion yield from BC (%)	70	
	Cost of BC contained in NCs (€/g BCNCs)	0.371	11
	Purification cost (€/g BCNCs)	2.882	89
Total cost BCNCs: cost of BC + BCNCs production (€/g BCNCs)		3.253	100

As regards the production of Sakacin-A, according to experimental evidence, CWP culture medium allows for production cost of Sakacin-A of around 5.67 €/10^6^ AU; the main part (91%) of the total production cost is due to purification, whose magnitude is associated to the use of the chromatographic column. Indeed, using the same column for repeated purifications (or for higher volumes of supernatant) would decrease purification cost by several order of magnitude. As a result, the total lab-scale cost for Sakacin-A is 65.67 €/10^6^ AU.

The total cost of BC (0.26 €/g) is the sum of production and purification costs: the former is mainly associated to β-galactosidase, while purification cost to NaOH. Purification contributes to 88% of the total cost of BC. Such value depends on the lab-scale price of NaOH, which may sharply decrease when increasing quantities are bought.

The total cost of BCNCs (3.253 €/g) is the sum of costs associated to yield from BC and for purification. As for Sakacin-A and BC, the main part of total cost again is due to purification (89%), in particular for sulfuric acid, which again may take advantage of strong price discount with the production upscaling.

Lab scale costs of the two formulations set up to produce the active materials (Sakacin-A/HPC) and Sakacin-A/BCNCs conjugates are based on experimental evidence and shelf-life trials. Formulation’s costs are provided for A4 format sheet (Table 4, first row). The estimated cost for Sakacin-A active formulation is 1.045 €/A4 sheet and includes the cost for HPC (0.023 €/A4) needed to impart viscosity. The cost for Sakacin-A/BCNCs conjugate is 1.699 €/A4, in this case no HPC was added but the cost of BCNCs production has to be considered (0.676 €/A4).

**Table 4:** Simulation of change in active formulations costs for decreasing levels of purification costs.

Reduction in purification costs (%)	Cost of Sakacin-A coating formulation (€/A4 sheet)	Cost of Sakacin-A/BCNCs coating formulation (€/A4 sheet)
**0**	**1.045**	**1.699**
10%	0.951	1.538
20%	0.858	1.378
30%	0.764	1.218
40%	0.671	1.057
**50%**	**0.577**	**0.897**

Lab scale costs (first row) and simulated pilot/plant scale costs (last row) are denoted in bold.

Simulating cost reduction when moving from laboratory to pilot and plant scale is not straightforward^3^. On one side, estimated lab-scale costs do not include energy, labour and fixed costs associated to lab facilities; such omitted inputs would increase production costs at pilot and plant scale. The extent of such increase depends on the production scale (i.e. amount of output on which omitted costs are spread). On the other hand, moving to plant-level brings an abatement in average cost, for economies of scale. Such cost savings are due to technological (average fixed costs abatement) and market reasons (decreasing input prices for increasing input quantities). Interestingly, both effects may apply to the cost structure presented here. In particular, the main part of ingredient costs (Sakacin-A, BC and BCNCs) are associated to purification (90%). In the case of Sakacin-A, purification cost may decrease, according to experts’ experience, by one to two orders of magnitude, for the abatement of fixed cost of chromatographic columns. In the case of BC and BCNCs, purification costs may drop because of input prices decrease at plant level (NaOH for BC and sulfuric acid for BCNCs).

To give an estimate of costs saving due to economies of scale, Table 4 provides a simulation in cost reduction, for decreasing levels of purification costs. Even if purification cost for Sakacin-A can be reduced from 10 to 100 times, a maximum 50% reduction has been set prudentially, as the upscaling would imply additional costs (labour, energies, other fixed assets) not considered here.

It is therefore plausible to consider that as purification costs fall by 50%, Sakacin-A formulation would cost 0.577 €/A4 sheet, while Sakacin-A/BCNCs formulation would cost 0.897 €/A4 sheet (excluding energy, labour and fixed asset costs).

Such estimated pilot/plant level costs of the active formulations may represent a first step to assess the economic feasibility for their adoption by the food industry. Feasibility assessment should compare supply side (production costs) and demand side (consumer acceptance) features of active packaging formulations^50^. Form the demand side, the new package should be accepted by a certain share of consumers; furthermore, a minimum amount of them should be willing to pay (WTP) an additional price to cover active packaging costs. Evidence on consumer acceptance and WTP for smart/active packaging are scant and sensitive to packaging technology, food product and information on technology ^51^. Therefore, specific analysis should be carried out to assess WTP for active packaging formulations presented in this paper. Their economic feasibility would emerge by comparing consumer WTP with the additional price of food packaging due to active formulations.

## Conclusions

High costs and complex purification processes represent an obstacle in large scale utilization of bacteriocins as food preservatives. However, their application in active packaging may represent a promising solution to enhance food safety in ready-to-eat products and to control food spoilage, fulfilling the consumers request for minimally processed food containing natural additives.

A promising approach to reduce production costs is the use of cheap by-products, such as CWP characterized by high disposal costs and environmental impact, as substrate for producing the molecules involved in the active packaging. Furthermore, this approach adds the value of carrying out a practical example of a circular economy production procedure by using a food industry by-product to produce antimicrobials and bacterial cellulose for food preservation.

In the present paper indeed, we provide a straightforward process to obtain BCNCs conjugated with the bacteriocin Sakacin-A starting from CWP, and their application into an active food packaging. The effectiveness of the antimicrobial packaging has been proven both in vitro and in real food intentionally inoculated with *Listeria*, demonstrating the valid approach of limiting the risk of *L. monocytogenes* outbreaks in foodstuff.

With regard to the economic feasibility of the process, even if under a cost–benefit viewpoint conjugation of the bacteriocin with BCNCs may appear as a process not providing an additional value to the product, compared to the use of the sole Sakacin-A, conjugates are plenty of promising, still unexplored properties. In detail, in addition to the widely studied and well-known effects of BCNCs on specific properties of the packaging (e.g. oxygen and water permeability), our results suggest that conjugation could modulate the kinetic of the antimicrobial molecule release from the packaging to the food. Addition of Sakacin-A/BCNCs conjugates to food packaging indeed, represents a promising strategy to maximize the cost-performance ratio intrinsically connected with the application of this kind of expensive compound, fully exploiting their functional properties.

Future studies will be dedicated at modulating the release kinetic of the antimicrobial compound considering the physico-chemical properties of food (humidity, pH, centesimal composition, etc.), and their effects on the technological properties of the packaging. These results should also be complemented with a sensory analysis of the cheese samples, which would help to evaluate the eventual occurrence of off-flavors derived from peptides to the cheese surface.

## Methods

### Materials

All chemicals were reagent grade purchased from Merck KGaA (Darmstadt, Germany) except for lecithin, sodium thiosulfate and l-histidine from Carl Roth GmbH (Karlsruhe, Germany), and hydroxypropyl cellulose (Klucel) from Ashland Inc. (Covington, USA). β-galactosidase (Lactozyme 2600 L) was from Novozymes (Bagsvaerd, Denmark), whereas cellulase (*Trichoderma reesei*) from Merck. Biolife Italiana Srl (Milan, Italy) supplied all culture media ingredients and Agar *Listeria* Ottaviani Agosti (ALOA) with the dedicated enrichment and selective supplements; De Man, Rogosa and Sharpe (MRS) Broth was from Scharlau Chemie (Barcelona, Spain), Tryptic Soy Broth (TSB) from Merck. CWP was kindly supplied by Latteria Soresina (Soresina, Italy): a single batch was used throughout all the project (lactose 4.7%, protein 0.01%, dry residue 5.5%, ash 0.6%).

### Microbial strains

*Komagataeibacter xylinus* DSM 2325 (DSMZ: Deutsche Sammlung von Mikroorganismen und Zellkulturen GmbH, Braunschweig, Germany) was used for BC production, maintained on GYC medium (g/L): glucose 50, yeast extract 10, CaCO_3_ 30, agar 15, pH 6.3, sterilization 121 °C for 15 min, formulated from single components in distilled water.

*Lactobacillus sakei* DSMZ 6333 was used for Sakacin-A production, *L. innocua* DSMZ 20649 as indicator strain, maintained respectively in MRS and TSB broth. Strains were stored at − 80 °C in appropriate medium (GYC for *K. xylinus*, MRS for *L. sakei* and TSB for *L. innocua*) with 50% glycerol and cultures propagated twice before use.

### BC production, purification and characterization

Seed cultures were prepared inoculating a single colony in Hestrin–Schramm (HS)^52^ medium (g/L): glucose 20, yeast extract 5, tryptone 5, Na_2_HPO_4_ 2.7, citric acid 1.15, pH 6.0, sterilization 121 °C for 15 min, supplemented with 2 U/mL cellulase (sterile-added after sterilization from a 200 U/mL sterile solution in distilled water); growth (20 mL of medium in 100 mL Erlenmeyer baffled flask) was performed for 24 h at 30 °C on orbital shaker (150 rpm). The whole culture was used to inoculate 100 mL of the same medium in 1000 mL Erlenmeyer baffled flask; after growth, cells were harvested by centrifugation (5000×*g* for 15 min at 25 °C), washed once with deionized water and used for the inoculation of the production vessel.

BC production was conducted in static fermentations using (25 × 25) cm polypropylene vessels, containing 1 L of CWP supplemented with β-galactosidase (0.5 U/mL); BC accumulated as a thin film on the surface of the medium. After 7 days at 30 °C pellicles were harvested, washed twice with deionized water, boiled (30 min in 0.5 M NaOH), and washed with deionized water until neutral pH; finally, BC sheets were dried at 50 °C until constant weight. Fermentation batches were performed in triplicate.

BC structural organization was investigated by XRD using a Bruker D2 Phaser diffractometer (CuKα radiation, Theta/Theta geometry, spinner, and LynxEye linear PSD detector): 30 kV, 10 mA, 2θ range 6°–50°, step size 0.02°, time per step 2 s, sample rotation 15 rpm, detector opening of 5° 2θ. X-ray patterns were evaluated using Bruker EVA v14.2 software (Bruker AXS, 2008). BC profiles were compared with the idealized powder diffraction patterns for cellulose polymorphs^39^.

### BCNCs production and characterization

A stock dispersion of BC 12% (w/w) in distilled water was prepared; 1 g of this dispersion was added in 14 g of 65% H_2_SO_4_, stirring at room temperature with an Ultra-Turrax T25 basic (Ika-Werke, Stanfen, Germany) at 8000 rpm for 5 min, and then incubating at 55 ± 1 °C for 2 h under gentle stirring (400 rpm). 14 mL of distilled water were then added and centrifuged at room temperature at 5920×*g* for 20 min. BCNCs collected at the bottom of the tube were washed twice with distilled water (30 mL) and then dialyzed in cellulose-based dialysis tube (cut-off 12000 Dalton, Merck) up to neutral pH. BCNCs yield was assessed gravimetrically through a halogen-lamp moisture content analyzer HS43S-MC (Mettler Toledo, Greifensee, Switzerland) at 105 °C.

BCNCs stability was investigated by electrophoretic light scattering (ELS) using a LitesizerTM 500 (Anton Paar, Rivoli, Italy) system, allowing the measurement of the ζ-potential at pH 7. Size distribution was obtained through a DLS Nanotrac Flex In-situ Analyzer (MicroTrac GmbH, Krefeld, German), with a stabilization time of 60 s, using viscosity and refractive index of water of 0.8872 cP and 1.33, respectively. The concentration of BCNCs in both ELS and DLS measurements was 0.1%. TEM was performed using a LEO 912 AB energy-filtering transmission electron microscope (EFTEM) (Carl Zeiss, Oberkochen, Germany) operating at 80 kV, equipped with a ProScan 1 K Slow-Scan CCD camera (Proscan, Scheuring, Germany). Samples for TEM analyses were prepared by drop-casting few microliters of dispersion (0.05%) onto Formvar-coated Cu grids (400-mesh), leaving them (24 h) at room temperature to allow water evaporation.

### Sakacin-A production and enrichment

Cultures (7 L) of *L. sakei* were carried out in a fermenter (Omnitec Bio, Sedriano, Milano) as reported in Musatti et al*.*^3^. Sakacin-A was quantified in terms of anti-*Listeria* activity through the agar diffusion assay^31^. A preparation enriched in Sakacin-A was obtained by a reverse-phase traditional chromatography: culture was centrifuged at 5000×*g* for 30 min at 4 °C; aliquots of supernatant (750 mL) were loaded onto a chromatographic column packed with 10 g of PoraPak RXN RP (Waters, Sesto San Giovanni, Italy). The column was washed with 100 mL of trifluoroacetic acid (TFA) 0.1% to remove the unbound fraction (first wash), and then by 100 mL of ACN 25%, TFA 0.1% to elute less hydrophobic peptides (second wash). Elution of Sakacin-A was achieved with 100 mL of ACN 40%, TFA 0.1% (enriched sakacin-A). Second wash and enriched sakacin-A fractions were concentrated in Rotavapor (Buchi Italia, Cornaredo, Italy) to remove ACN, freeze-dried (Alpha 2–4 LD freeze-dryer, Christ, Osterode am Harz, Germany) and redissolved in 10 mL of milli-Q water. All eluted fractions were characterized in term of (1) anti-*Listeria* activity through the agar diffusion assay^33^, (2) protein content by the Lowry method, and (3) peptide pattern by reverse-phase-HPLC (RP-HPLC). RP-HPLC separations were run in a SIMMETRY300 C18 (5 μm) (4.6 × 250) mm column (Waters, Sesto San Giovanni, Italy) fitted on a chromatographic apparatus (Waters) composed by two 510 HPLC Pumps, a 717plus Autosampler and a 996 Photodiode Array Detector. Mobile phase was flown at 0.8 mL/min, mixing solutions A (TFA 0.1% in water) and B (TFA 0.1% in ACN) as follows: 10 min isocratic 100% solution A, 40 min linear gradient to 40% solution A and 60% solution B. Sakacin-A-containing preparation (i.e. freeze-dried enriched Sakacin-A redissolved in milli-Q water) was stored a − 20 °C until use.

### Preparation of Sakacin-A/BCNCs conjugates

Conjugation between Sakacin-A and BCNCs was achieved exploiting their opposite charge. A suitable volume of Sakacin-A-containing preparation (typically around 6600 AU/mL) was diluted 50 fold in water, mixed with BCNCs suspension (ratio 75 AU/mg BCNCs) and incubated at room temperature, 90 rpm (Shaker stirrer F340, Falc, Treviglio, Italy) for 10 min. Sakacin-A/BCNCs conjugates were separated by centrifugation at 13000×*g* for 10 min (the net weight of the conjugates was approximated to the weight of the BCNCs used, given the negligible amount of the adsorbed protein fraction, see results). The supernatant solution (unbound fraction) was characterized by activity test and RP-HPLC.

Conjugates stability was assessed by incubating 1 mg of them in 1 mL of water at pH 5 and pH 11 (pH was adjusted with HCl and NaOH, respectively), and of NaCl 20 mM. After 10 min incubation at room temperature, 90 rpm, samples were centrifuged at 13000×*g* for 10 min. The supernatant was subjected to activity test and RP-HPLC to look for released activity.

### Production and characterization of coated paper samples

Four coating formulations were used to produce the coated paper samples, as follows: (1) Sakacin-A/BCNCs conjugates (73 mg/mL conjugates, providing 5500 AU/mL); (2) BCNCs (73 mg/mL BCNCs); (3) Sakacin-A (18.4 mg/mL Sakacin-A-containing preparation, providing 5000 AU/mL, HPC 73 mg/mL; Sakacin-A (concentration has been reduced to obtain more comparable coated activity on paper samples); (4) HPC as control (HPC 73 mg/mL). HPC was added as thickener only in samples not containing BCNCs.

Coatings were performed using an automatic film applicator Sheen 1137 (Sheen Instruments, Cambridge UK), equipped with a Meyer bar type 1120/25/50, traverse speed of 100 mm/s. The paper substrate was cellulose Kraft white paper 45 g/m^2^, one side machine glazed, wet strength, normally used as fresh food wrapping paper. A fixed amount of coating formulation (2.5 mL) was laid on the glazed side of paper sheets (effective coated surface area about 500 cm^2^) achieving an antimicrobial activity of about 25 AU/cm^2^. Coated paper sheets were dried for 5 min at 50 °C and then conditioned in a standard atmosphere (23 °C, 50% R.H.) before testing. Coated paper sheets were characterized in term of grammage^53^, thickness^54^, surface roughness^55^, and tensile strength^56^. Surface wetting was analyzed by optical contact angle using an OCA 15 plus (Data Physics GmbH, Germany), dispersing a water drop volume (4.0 µL) by the sessile drop method. Grease resistance was determined by surface repellency test (Kit Test) according to ^57^.

### In vitro determination of antimicrobial activity of the coated paper samples

Antimicrobial efficacy of the active paper samples was evaluated by two in vitro tests. A liquid pre-culture of this microorganism in TSB, incubated for 16 h at 37 °C (final cell concentration (5 × 10^8^ cells/mL), was used as inoculum for the reported trials.

As regards the MIC determination, the assay was carried out measuring the turbidity (OD) of a *L. innocua* TSB liquid culture (inoculum 1% v/v from an overnight pre-culture in the same medium) incubated at 37 °C up to 24 h, employing a PowerWave XS2 Microplate Spectrophotometer (BioTek, USA) at 600 nm (measures taken every 15 min). The Sakacin-A containing preparation or the conjugates were suspended in TSB and filter-sterilized at a concentration range of 1–800 AU/mL. The MIC was defined as the lowest concentration able to produce inhibition of *Listeria* growth.

Qualitative assessment of antimicrobial activity was carried out applying the agar disk diffusion assessment (official method BS EN 1104)^58^: *Listeria* pre-inoculated TSA plates were prepared by adding 1% (v/v) of the *Listeria* preculture in melted TSA (approximately 10^6^ cells/mL): plates were then left to solidify under the sterile biosafety hood. Paper portions (20 mm diameter) were subsequently placed onto the surface of the TSA solid cultures in plates, that were incubated for 16 h at 37 °C. The antimicrobial efficacy was determined measuring the diameter of the clear halo of *Listeria* growth inhibition under and around the paper (mm).

For quantitative assessment, the official AATCC 100/1998 procedure was followed^59^: paper portions (2.5 × 2.5 cm^2^) were placed into empty Petri plates, and 200 µL of a *Listeria* cell suspension prepared at a concentration of 5 × 10^5^ cells/mL in 20% TSB culture medium (1 volume of TSB in 4 volumes of sterile isotonic solution) were poured onto each paper sample. Plates containing the materials were then incubated for 24 h at 37 °C; bacteria were subsequently resuspended from the paper samples with 50 mL of neutralizing solution (g/L): lecithin 3, sodium thiosulfate 5, L-histidine 1, Tween-80 30, potassium phosphate buffer 0.3 mM pH 7.2 ± 0.2 10 mL. These suspensions were decimally diluted and pour-plated in ALOA selective medium for *Listeria* determination. Plates were incubated at 37 °C for 48 h and counts reported as logarithm of the number of colony forming units (Log CFU/mL), and mean and standard deviation calculated. Experiments were performed in triplicate.

### Determination of antimicrobial activity on fresh cheese

Soft cheese known as “stracchino” (composition: milk, salt, rennet; fat on dry matter 52% min) was purchased from a large-scale retail channel (Carrefour, Milano), aseptically cut into sections of 5 × 5 × 1 cm^3^ (approximately 20 g) and surface inoculated with a *L. innocua* cells suspension prepared diluting the already described pre-culture to obtain a total microbial load of 10^3^ cells/g. Inoculated samples were left 30 min under the safety hood to allow *Listeria* to absorb onto cheese matrix. Samples were then wrapped with paper samples differently coated (50 cm^2^): Sakacin-A/BCNCs conjugates (25 AU/cm^2^), Sakacin-A (25 AU/cm^2^), BCNCs and HPC reference. This last sample, together with unwrapped cheese were set as negative controls.

All cheese samples were packaged under modified atmosphere (MAP). Packaging was performed in 30 µm metalized oriented polypropylene (m-OPP, SAES Coated Films S.p.A, Roncello, Italy) pouches. Protective atmosphere (70% CO_2_: 30% N_2_) was generated with a gas mixer (Mod. 9000, PBI Dansensor, Ringsted, Denmark), fitted with a Multivac vacuum chamber machine (Wolfertschwenden, Germany). Packages were stored at 5 ± 1 °C for 21 days. At t_0_ and 21 days, cheese samples were transferred aseptically into Stomacher bags (VWR blender bag, Milano, Italy), filled with physiological solution (9 g/L NaCl, 9 × sample weight) and blended in a Stomacher (Star Blender LB 400, VWR, Milano, Italy) for 3 min. These suspensions were decimally diluted and pour-plated either in ALOA selective medium for *Listeria* determination and in TSA for the Total Aerobic Count (TAC). Plates were incubated at 37 °C (*Listeria*) or 30 °C (TAC) for 48 h. Counts were reported as logarithm of the number of colony forming units per unit of cheese weight (Log CFU/g), and mean and standard deviation calculated.

Experiments were replicated twice. Counts were reported as Log CFU/g cheese and mean and standard deviation calculated.

### Statistical analysis

One-way ANOVA and Tukey Test (*p* < 0.05) were used to compare data by using the software SigmaPlot 14.0 (Systat Software, Inc., San Jose, CA, USA).
